# Arterial spin labeling for presurgical localization of refractory frontal lobe epilepsy in children

**DOI:** 10.1186/s40001-021-00564-0

**Published:** 2021-08-06

**Authors:** Jia Zhang, Heng Zhang, Yang Li, Meng Yuan, Jinxiu Zhang, Huan Luo, Zeshan Yao, Jing Gan

**Affiliations:** 1 Department of Pediatrics, West China Second University Hospital, Sichuan University, No. 20, Section Three, South Renmin Road, Chengdu, 610041 China; 2 Key Laboratory of Obstetrics & Gynecologic and Pediatric Diseases and Birth Defects of the Ministry of Education, Sichuan University, Chengdu, Sichuan China; 3 Department of neurosurgery, West China Hospital of Sichuan University, Chengdu, 610041 China; 4 Anlmage Technology, Beijing, China

**Keywords:** Arterial spin labeling, Presurgical evaluation of epilepsy, Frontal lobe epilepsy, Drug-resistant epilepsy, Focal cortical dysplasia

## Abstract

**Background:**

Epilepsy is one of the most common chronic neurological diseases. Despite the great variety and prevalence of antiepileptic drug treatments, one-third of epilepsies remain drug resistant. The frontal lobe is extensive, and frontal lobe seizures are difficult to locate, which increases the difficulty of the preoperative localization of the epileptogenic zone.

**Case presentation:**

Two previously healthy girls with refractory frontal lobe epilepsy showed significant perfusion abnormalities in the right frontal lobe using the cerebral blood perfusion (CBF) quantitative analysis system. They became seizure-free after lesionectomy of the frontal lobe by ASL combined with electroencephalography (EEG) rapid localization. The histopathological diagnosis was focal cortical dysplasia (FCD) type IIa and IIb.

**Conclusions:**

The positive outcome suggests that the combined use of ASL with EEG could be a beneficial option for the presurgical evaluation of pediatric epilepsy.

**Supplementary Information:**

The online version contains supplementary material available at 10.1186/s40001-021-00564-0.

## Background

Epilepsy is one of the most common chronic neurological diseases and has a prevalence rate between 0.8 and 1.2%. Despite the great variety and prevalence of antiepileptic drug treatments, one-third of epilepsies remain drug-resistant, with epilepsy surgery accounting for 10–50% of these patients’ subsequent treatment options [1]. Presurgical evaluation of these patients with drug-resistant epilepsy is very important. While MRI is a useful tool for the identification of epileptogenic structures, in the presurgical evaluation of pediatric epilepsy, 30–40% of patients can have a negative MRI finding [2]. Noninvasive or invasive examinations, such as PET (positron computed tomography), SPECT (single-photon emission computed tomography), MEG (magnetoencephalography), SISCOM (subtraction ictal single-photon emission computed tomography coregistered to MRI), and SEEG (stereotaxic electroencephalography), are the preferred presurgical evaluations of epilepsy. Nevertheless, each of them has shortcomings and limitations.

Frontal lobe seizures often result in rapid synchronous electrical discharge from bilateral frontal lobes, and they are difficult to interpret due to artifacts. Scalp EEG has difficulty locating the foci, which increases the difficulty of preoperatively locating the epileptogenic zone. Arterial spin labeling (ASL), as a functional magnetic resonance imaging (fMRI) technique, exploits variations in blood oxygen levels to detect changes in cerebral hemodynamics and locate the epileptogenic zone without contrast agents and radiation. ASL has the advantages of being fast, convenient, repeatable, nonradiogenic, and relatively simple. However, at present, few studies have reported the role of ASL in the localization of epileptogenic regions in temporal lobe epilepsy. In this paper, the application of ASL in the presurgical evaluation of pediatric epilepsy is successfully illustrated through two cases of pediatric drug-resistant frontal epilepsy who became seizure-free via surgical resection of epileptogenic regions localized by ASL. Thus, this technique could play an important role in the presurgical evaluation of pediatric epilepsy.

## Case presentation

### Patient history

#### Case 1

A 4-year-old previously healthy girl was admitted to our hospital due to repeated seizures for 9 months. During the ictal phase, her seizures were characterized by emotional seizures with evolution to bilateral tonic–clonic seizures with impaired awareness that lasted for several minutes. Two days prior to admission, her condition worsened with more frequent seizures at up to 90 per day. On examination, she presented growth in the 90th percentile, normal neurodevelopment, and no developmental delays or regressions. No dysmorphic features, tremors, ataxia, or involuntary movements were observed. Findings on cranial nerve examination were normal. The patient was delivered by cesarean section at 37 weeks of gestation. There was no history of hypoxic asphyxia or postnatal resuscitation. Routine cerebrospinal fluid testing, autoimmune encephalitis-related antibodies, paraneoplastic syndrome-related antibodies, oligoclonal bands, aquaporin-4, myelin oligodendrocyte glycoprotein, glial fibrillary acidic protein, autoantibodies, thyroid-related antibodies, cardiolipin antibodies, antineutrophil cytoplasmic antibodies, metabolic screening, and other screening results were all negative. Levetiracetam (50 mg/kg.d), clonazepam (0.1 mg/kg.d), oxcarbazepine (40 mg/kg.d), and lacosamide (6 mg/kg.d) were successively administered. However, the outcomes were poor, as seizure control was not obtained from any of these treatments.

#### Case 2

A 7-year-old previously healthy girl was admitted to our hospital due to repeated seizures for 4 years. The main manifestation was focal seizures or focal to generalized tonic-clonic seizures. Oxcarbazepine (30 mg/kg.d), sodium valproate (30 mg/kg.d), levetiracetam (20 mg/kg.d), and lacosamide (6 mg/kg.d) were successively administered with poor outcomes, and seizure control was not obtained from any of these treatments. Two days before admission, she presented with panicking, screaming, and babbling accompanied by dizziness and headaches after the emotional outburst, which lasted approximately half an hour. On examination, her neurodevelopment was normal and she did not present developmental delays or regressions. No dysmorphic features, tremors, ataxia, or involuntary movements were observed. Cranial nerve examination findings were normal. She was delivered at 32 weeks of gestation and had a birth weight of 1350 g. There was no history of hypoxic asphyxia or postnatal resuscitation. The results of routine cerebrospinal fluid tests; tests for autoimmune encephalitis-related antibodies, paraneoplastic syndrome-related antibodies, oligoclonal bands, aquaporin-4, myelin oligodendrocyte glycoprotein, glial fibrillary acidic protein, and autoantibodies; metabolic screening tests; and other screening tests were all negative.

### EEG

Routine EEG and video EEG (VEEG) displayed epileptiform discharges of the right frontal lobe in both girls. VEEG of case 1 showed interictal slow background activity with low-high amplitude sharp and sharp-slow waves and 1.5–3 Hz polymorphic slow waves in the right or bilateral forehead, frontal lobe regions, and anterior temporal regions during wake-up and sleep, with low-amplitude sharp waves presenting in an isolated way or continuously and potentially spreading to adjacent leads or all leads (Additional file 1: Figure S1). During ictal onset, four clinical seizures were identified that were characterized by sudden explosive screams and deviation of both eyes to the right, followed by generalized tonic–clonic seizures for 20–30 s (Additional file 1: Figure S2). The synchronous EEG displayed right frontal epileptiform discharges that rapidly spread to the bilateral frontal area with low-medium amplitude sharp wave rhythms or sharp-slow waves, followed by generalized low-medium amplitude slow waves mixed with low-amplitude fast wave rhythms. In case 2, VEEG showed multiple spike (slow) waves in the right frontal, frontal, and anterior temporal regions (Additional file 1: Figure S3).

### MRI

**Fig. 1:**
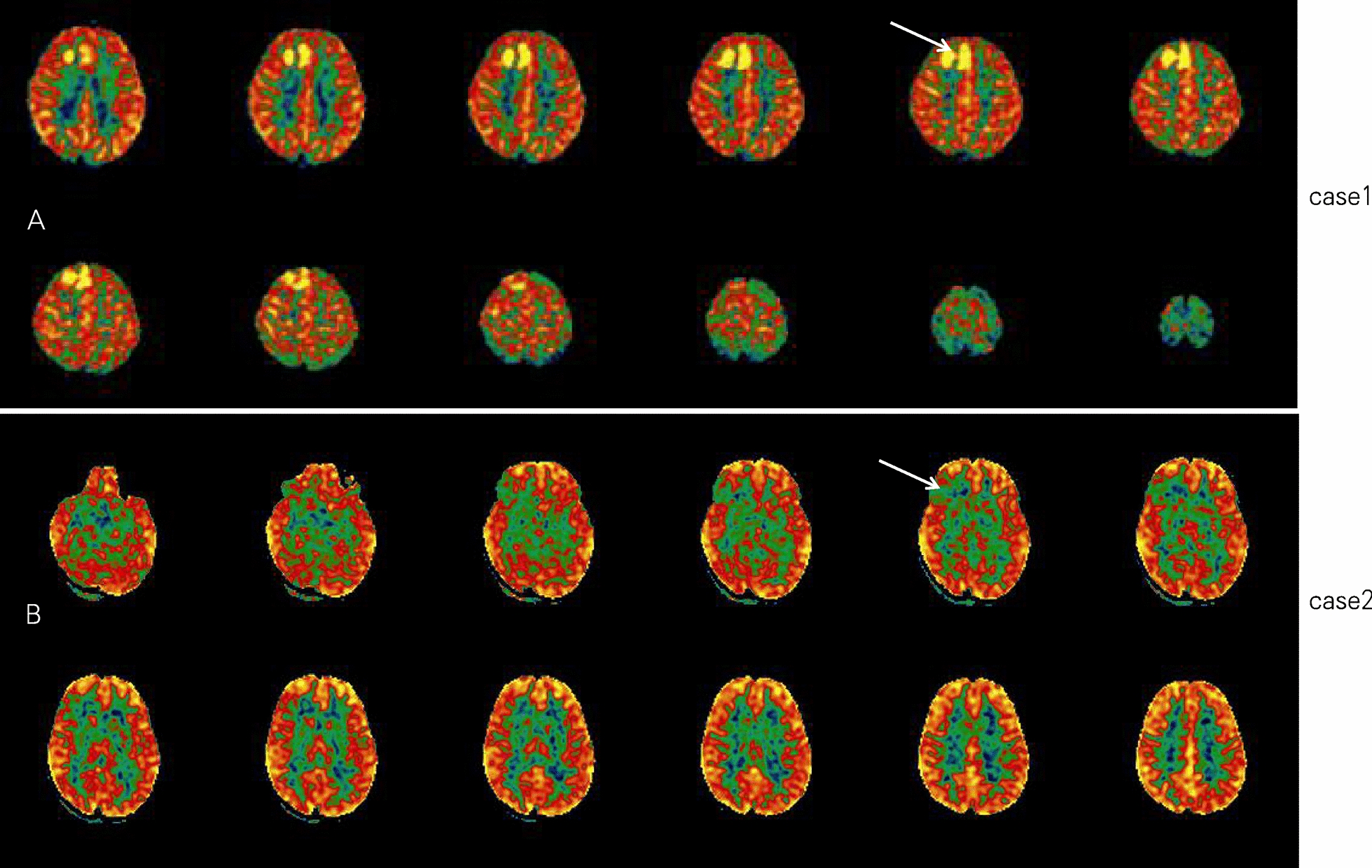
Preoperative ASL. **A** Case 1: ASL perfusion MRI depicts two hyperperfusions in the right medial frontal lobe, which is consistent with the epileptiform discharge position detected by VEEG and the suspicious thickened areas in FLAIR sequences. **B** Case 2: hypoperfusion was observed in the right frontal cortex, and the difference between the left and right frontal lobes exceeded the normal range of 0.2

Preoperative MRI was performed with a GE Sigma 1.5T superconducting MR scanner. The cranial scan was performed with the head orthogonal coil, T1WI, T2WI, and fluid attenuation inversion recovery (FLAIR) sequence. T1WI used a spin-echo sequence of TR: 500 ms and TE: 15. T2WI used a fast spin-echo sequence of TR: 3000 ms and TE: 90 ms. FLAIR used a sequence of TI: 2200 ms, TR: 8000 ms and TE: 140 ms. 3D ASL used a sequence of TR, TE, and PLD at 1025 ms. Head MRI in both girls suggested a suspicious signal abnormality and the possibility of dysplasia of the local right frontal cortex (Additional file 1: Figure S4). Considering that the patient’s seizures were drug-resistant, it was important to identify the responsible lesion and localize the lesion as soon as possible before surgical treatment. Therefore, 3D ASL cerebral perfusion imaging was performed to locate the lesion responsible for epileptic activity. The examination revealed abnormal perfusion areas in the right medial frontal lobe (Fig. 1A) using a cerebral blood perfusion quantitative analysis system. PLD (1025 ms) imaging demonstrated that the mean value of cerebral blood perfusion (CBF) of the lesion was 119.14 ml/100 g/min in case 1, which was + 217% of the mean value of the whole brain on the right hemisphere, presenting abnormally high perfusion. Case 2 had hypoperfusion in the right frontal cortex, and the difference between the left and right frontal lobes exceeded the normal range of 0.2 (Fig. 1B). Both of these results were consistent with the abnormal discharge position detected by EEG. In addition, the 3D ASL sequence demonstrated that the two abnormal perfusion areas in the right frontal cortex corresponded to the suspicious thickened areas in the MRI sequences.

### Surgery and postoperative period

**Fig. 2:**
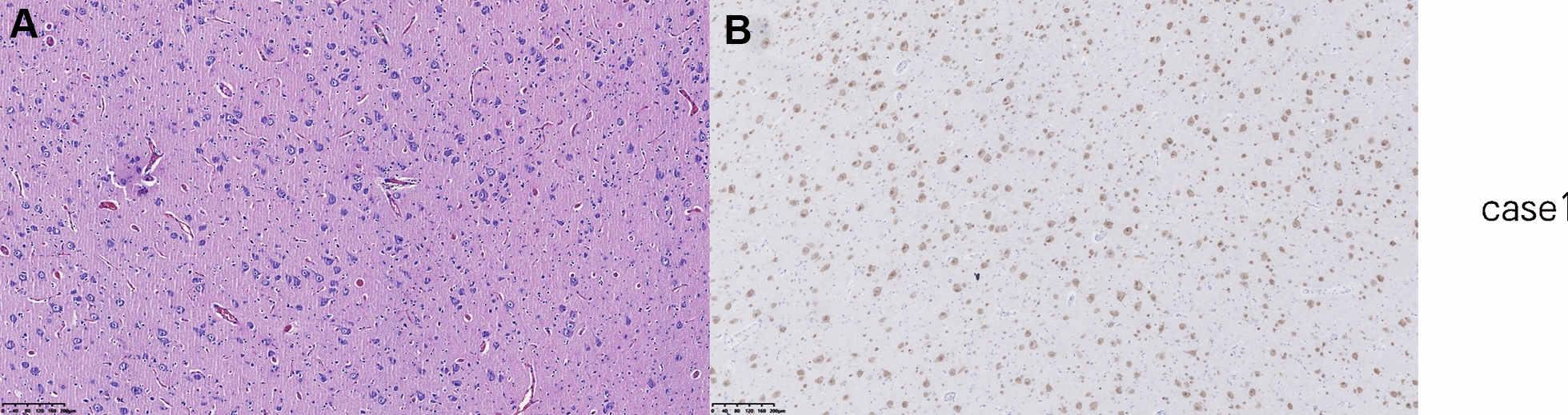
Postsurgical histopathology. Histopathological diagnosis was FCD type IIa with dyslamination and dysmorphic neurons in case 1 (**A**) and FCD type IIb with dysmorphic neurons and balloon cells in case 2 (**B**)

**Fig. 3:**
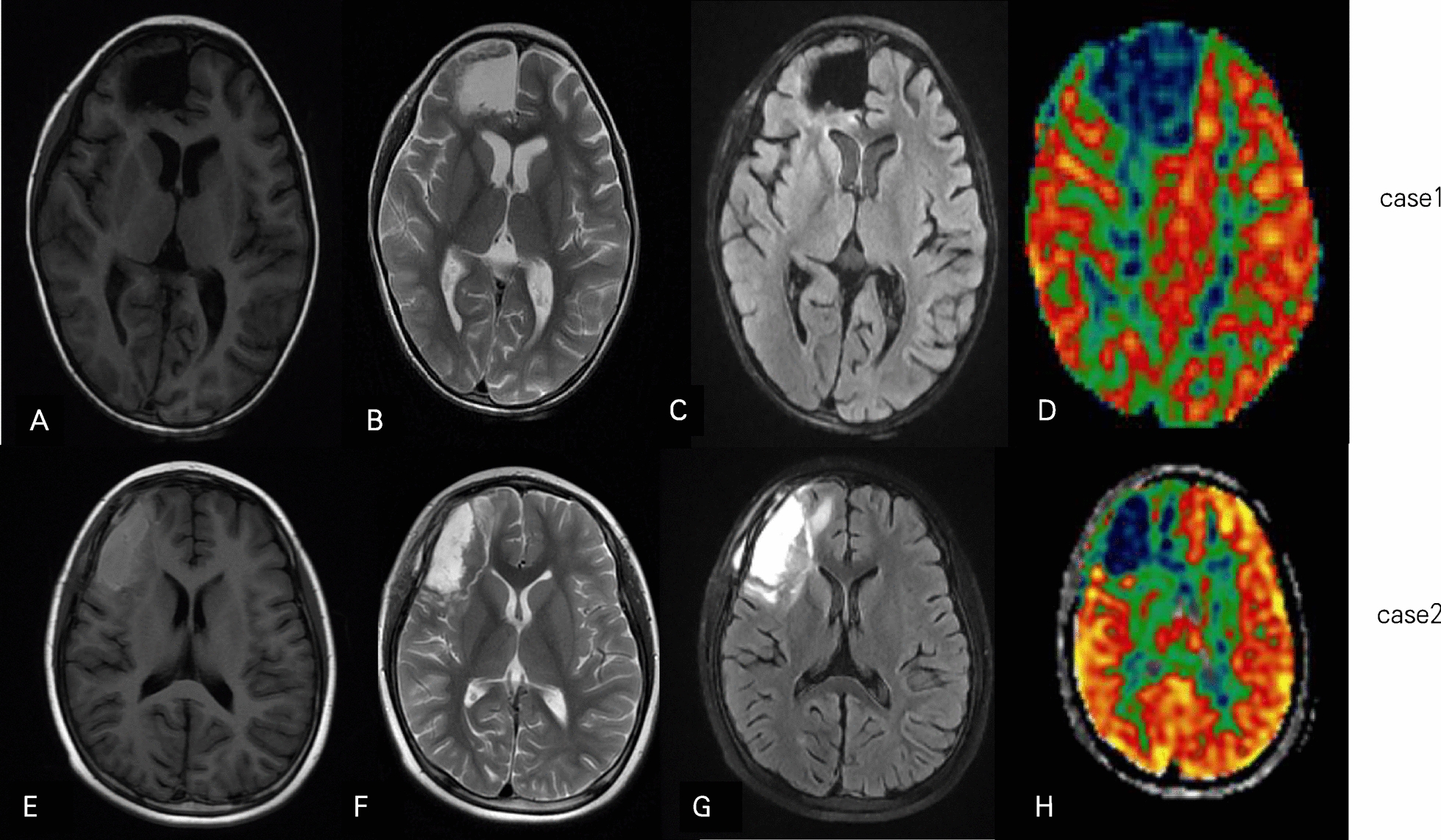
**A**–**H** Postoperative MRI and ASL imaging of both cases demonstrated that there were abnormally hypoperfusion areas in the right medial frontal lobe

According to the characteristics of the patient’s clinical symptomology and results from VEEG, MRI, and ASL scans, the epileptic lesions for both cases were identified in the right frontal lobe. The range of resection was determined by intraoperative EEG monitoring with electrocorticography. Consequently, “epileptic foci resection” was performed. These patients became seizure-free following lesionectomy. Histopathological diagnoses were focal cortical dysplasia (FCD) type IIa and IIb (Fig. 2A, B). Postoperative head MRI and ASL of both girls (Fig. 3A–H) showed that there were abnormal hypoperfusion areas in the right medial frontal lobe. Using the quantitative analysis system of CBF, the imaging with PLD (1025 ms) presented a mean CBF value of 17.81 ml/100 g/min for the lesion, which was 36% of the average value of the entire right hemisphere, thus indicating abnormally significant hypoperfusion. The postoperative VEEG of case 1 (Additional file 1: Figure S5) displayed slow waves in the left frontal and anterior temporal regions. The postoperative VEEG of case 2 showed occasional sharp waves in the forehead and frontal regions (Additional file 1: Figure S6). In case 1, antiepileptic levetiracetam treatment was continued after the operation, and no recurrence of the seizure was observed for 6 months. In case 2, antiepileptic oxcarbazepine and sodium valproate treatment was continued after the operation, and no recurrence of the seizure was observed for 2 months. There was no significant difference in orientation, comprehension, cognitive function, mentality, memory, or daily living ability between the preoperative and postoperative tests. Moreover, the motor and sensory functions did not appear to be affected.

## Discussion

Frontal lobe epilepsy is characterized by various types of seizures depending on the area of the frontal lobe involved, and it often requires surgical management, especially for patients with focal cortical dysplasia (FCD). Consequently, the identification of techniques to locate the lesions before surgery has become the key to presurgical evaluation. Video EEG and head MRI are the most common investigation methods to be carried out in the preoperative diagnosis and localization of frontal lobe epilepsies, which do not always yield positive results, such as for many cryptogenic patients. Conventional MRI can provide clear and stereoscopic brain anatomy images and identify structural abnormalities in the brain; thus, it is an effective method for imaging examinations of structural epilepsy. However, in the presurgical evaluation of pediatric epilepsy, the occurrence of negative findings in MRI can be as high as 30–40% [2], whereas this value is 15–30% for adults with refractory focal epilepsy [3]. Therefore, it is essential to find ways to improve the positivity rate of MRI in patients with refractory focal epilepsy for presurgical evaluation.

ASL is a kind of functional MRI (fMRI) technique that reflects tissue perfusion with magnetic markers of water protons within the arterial blood as an endogenous tracer. ASL technology collects the labeled image and the reference image separately and quantitatively calculates the perfusion by subtracting the two images. ASL can obtain cerebral blood flow (CBF) perfusion images without the need for exogenous contrast agents or tracers, which is extraordinarily suitable for patients who require repeated examinations, follow-ups, and posttreatment evaluations [4]. Currently, it is mainly applied for the diagnosis of neurovascular diseases, brain abscesses, infections, inflammation, trauma, brain function diseases, neurodegenerative diseases, etc. ASL has great advantages in pediatric applications because children have higher brain water content and hematocrit than adults, which increases the number and longevity of tracers and thus the signal-to-noise ratio (SNR). Additionally, due to the incomplete gasification of the paranasal sinuses in children, the susceptible artifacts in the skull base are reduced, thus improving the image quality [4].

ASL and PET are consistent in the evaluation of patients with epilepsy, especially in MRI-negative patients [5]. Aboelsafa et al. reported a prospective study on ASL in identifying epileptic areas in MRI-negative patients. ASL was used to calculate the asymmetry index percent (AI%) and the percent asymmetry factor (AF%) of different regions on both sides of the brain and showed accuracies of 95.78% and 98.14%, respectively, and ASL combined with MRS achieved 100% sensitivity, 98.45% specificity, and 98.86% accuracy [6]. Nevertheless, at present, the actual application of ASL for the presurgical evaluation of epilepsy is still rare. Several studies have reported the localization value of ASL, such as in MRI-negative children with new onset seizures, mesial temporal lobe epilepsy, nonlesional focal impaired awareness seizures, intractable epilepsy, partial epilepsy status, and localizing the seizure onset zone [6–12].

Epilepsy can induce cortical hyperperfusion during seizures, especially following repeated seizures or status epilepticus. The relative hypoperfusion that occurs during the interictal phase may indicate local brain atrophy and gliosis. Studies have shown that the epileptogenic zone can be hyperperfused during the ictal phase, and the high signal gradually becomes a low signal after one week [9]. It may be that the hyperexcitability of neurons in the epileptic area at the early stage of epilepsy results in metabolic and functional changes leading to increased local CBF. With the prolonged course of the disease, local cerebral blood flow may be reduced due to the loss of local neurons, which suggests that ASL has potential advantages for follow-up evaluations of epilepsy [7]. Although current reports of hyperperfusion acquisition time during an episode are inconsistent, it may last for hours or even days. Lee et al. reported that ASL perfusion changes were observed 2 h to 90 days after seizure and remained excessive within 1 day after the seizure ended [8]. Changes in the cerebral blood flow state may be affected by multiple factors, including the cerebral perfusion pressure, carbon dioxide level, type of structural abnormalities, duration of seizures, seizure frequency, and epileptic focus. In our reports, case 1 underwent an ASL examination immediately after repeated seizure episodes. The findings suggested that there were significant hyperperfusion changes in the epileptogenic lesions, which was consistent with the clinical symptoms and VEEG. These results may have been caused by abnormal changes in the metabolism and function of neuronal overexcitement during an epileptic seizure that resulted in significantly increased CBF in corresponding brain regions. In case 2, the interictal ASL examination showed hypoperfusion in the focal zone, which is largely consistent with literature reports [9]. Both of these findings were consistent with clinical symptoms and VEEG.

Therefore, ASL can be used to locate epileptic lesions noninvasively in conjunction with EEG results. EEG has a certain diagnostic value for brain diseases, especially in patients with focal epilepsy, although it is limited to certain conditions and susceptible to various factors. In our two cases, ASL combined with EEG was used to accurately identify the epileptogenic zone of the patient with DRE. Postsurgical ASL and EEG indicated that the lesion was resected, which was further validated by the fact that the patient was seizure-free after the lesionectomy. Furthermore, motor, sensory, cognitive, and language functions were not affected. These results lay a foundation for the identification and accurate localization of epileptic lesions through the use of ASL combined with EEG and suggest that ASL cerebral perfusion imaging can be an alternative to PET imaging and localization that has the added advantages of being safer, cheaper, and faster. Moreover, ASL is relatively stable and less affected by the patient's natural state.

## Conclusions

In recent years, ASL has emerged as a new magnetic resonance imaging method for measuring cerebral blood flow, and it is characterized by its simplicity, nonradiogenicity, high spatial resolution, and good reproducibility. ASL has been successfully applied in clinical studies of many types of diseases; however, it is rarely used to assist in localizing epileptogenic regions in MRI-negative children with focal epilepsy. To the best of our knowledge, this is the first case report of the use and consequent analysis of the ASL technique in identifying epileptogenic regions in pediatric frontal lobe epilepsy patients. We believe that the experience of our two cases would encourage a larger use of this convenient method for the presurgical study of cortical epileptogenic lesions in epilepsy.

## Limitations

First, the number of cases in this study was limited. Second, we did not compare the sensitivity of ASL with that of ictal/interictal PET because PET is currently the most accepted standard for the preoperative evaluation of epilepsy surgery. Third, the range of resection was determined by intraoperative EEG monitoring with electrocorticography but not stereoelectroencephalography. More prospective studies in a larger number of patients are required to determine the location of ASL in epileptogenic lesions, especially in pediatric patients.

## Supplementary Information

**Additional file 1: Figure S1. **Preoperative interictal VEEG of case 1, displaying interictal slow background activity with sharp slow waves dominating in sleep, which can spread to adjacent leads or all leads. **Figure S2.** Preoperative ictal VEEG of case 1, showing epileptiform discharge in the right frontal area that rapidly spread to the bilateral frontal area. Extensive slow waves continued to discharge after the seizure. **Figure S3.** Preoperative VEEG of case 2. The ictal VEEG shows multiple spick (slow) waves in the right frontal, frontal, and anterior temporal regions. **Figure S4.** Preoperative head MRI of case 1: T1WI (A) and T2WI (B) sequences showed suspicious signal abnormalities, with a subtly increased FLAIR (C) signal in the right frontal lobe (black arrows). Case 2: T1WI (D), T2WI (E), and FLAIR (F) sequences showed suspicious signal abnormalities and the possibility of dysplasia of the local right frontal cortex. **Figure S5.** Postoperative VEEG of case 1, displaying slow waves in the left frontal and anterior temporal regions. **Figure S6.** The postoperative VEEG of case 2 showed occasional sharp waves in the forehead and frontal regions.

## Data Availability

The datasets used and/or analyzed during the current study are available from the corresponding author on reasonable request.

## Abbreviations

ASL: Arterial spin labeling
EEG: Electroencephalogram
PET: Positron computed tomography
SPECT: Single-photon emission computed tomography
MEG: Magnetoencephalography
SISCOM: Subtraction ictal single-photon emission computed tomography coregistered to MRI
SEEG: Stereotaxic electroencephalography
fMRI: Functional magnetic resonance imaging
VEEG: Video EEG
FCD: Focal cortical dysplasia
FLAIR: Fluid attenuation inversion recovery sequence
CBF: Cerebral blood perfusion
SMA: Supplementary motor area
AEDs: Anti-epileptic drugs
