# Supplementary material for: Arterial spin labeling for presurgical localization of refractory frontal lobe epilepsy in children
Source: Eur J Med Res. 2021 Aug 6;26:88. doi: 10.1186/s40001-021-00564-0 (PMC8349087; doi:10.1186/s40001-021-00564-0)
Supplement: Supplementary file 1 — Additional file 1: Figure S1. Preoperative interictal VEEG of case 1, displaying interictal slow background activity with sharp slow waves dominating in sleep, which can spread to adjacent leads or all leads. Figure S2. Preoperative ictal VEEG of case 1, showing epileptiform discharge in the right frontal area that rapidly spread to the bilateral frontal area. Extensive slow waves continued to discharge after the seizure. Figure S3. Preoperative VEEG of case 2. The ictal VEEG shows multiple spick (slow) waves in the right frontal, frontal, and anterior temporal regions. Figure S4. Preoperative head MRI of case 1: T1WI (A) and T2WI (B) sequences showed suspicious signal abnormalities, with a subtly increased FLAIR (C) signal in the right frontal lobe (black arrows). Case 2: T1WI (D), T2WI (E), and FLAIR (F) sequences showed suspicious signal abnormalities and the possibility of dysplasia of the local right frontal cortex. Figure S5. Postoperative VEEG of case 1, displaying slow waves in the left frontal and anterior temporal regions. Figure S6. The postoperative VEEG of case 2 showed occasional sharp waves in the forehead and frontal regions. [file 40001_2021_564_MOESM1_ESM.pdf]

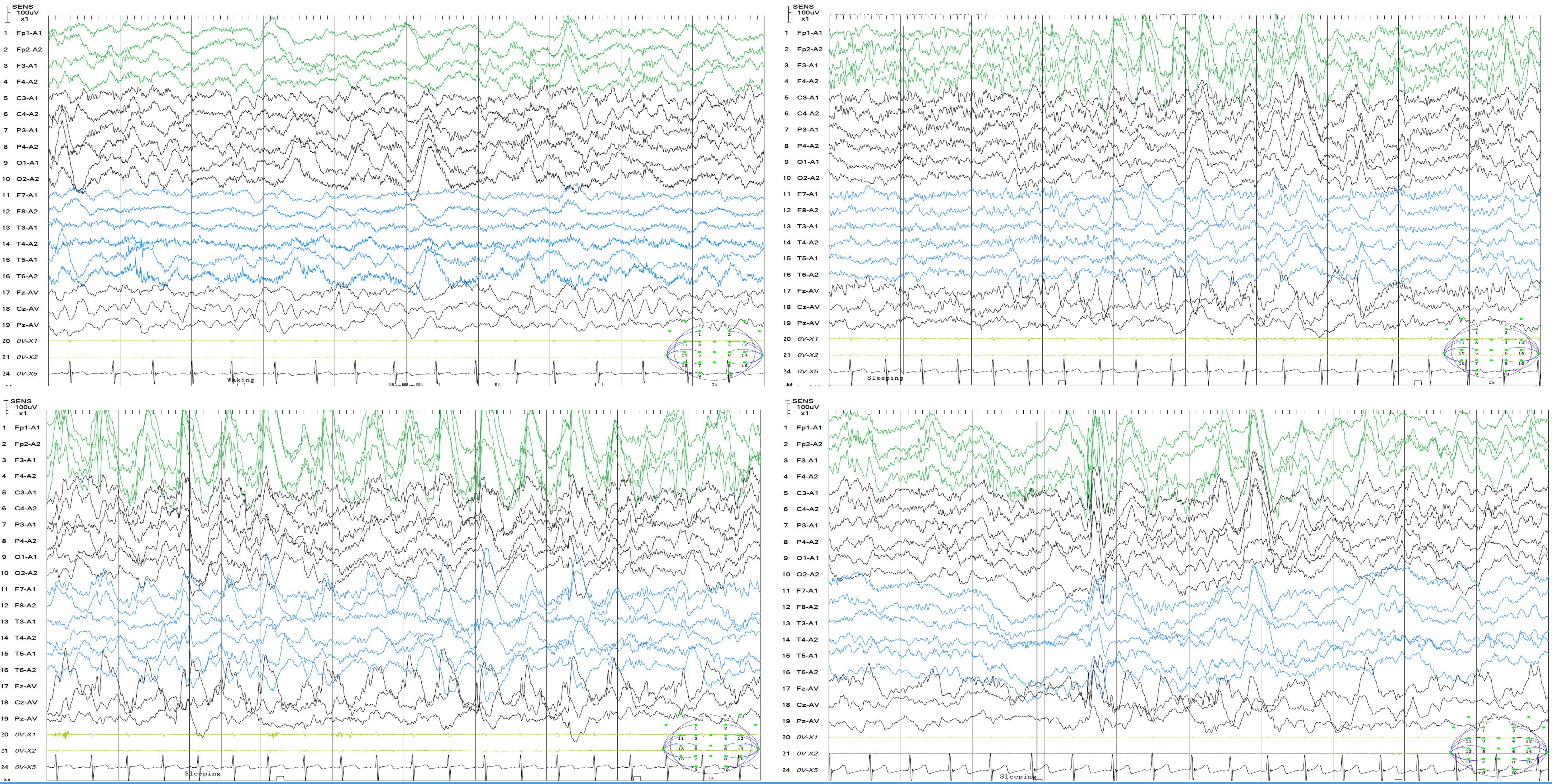


Supplementary figure 1: Preoperative interictal VEEG of case 1, displaying interictal slow background activity with sharp slow waves dominating in sleep, which can spread to adjacent leads or all leads.

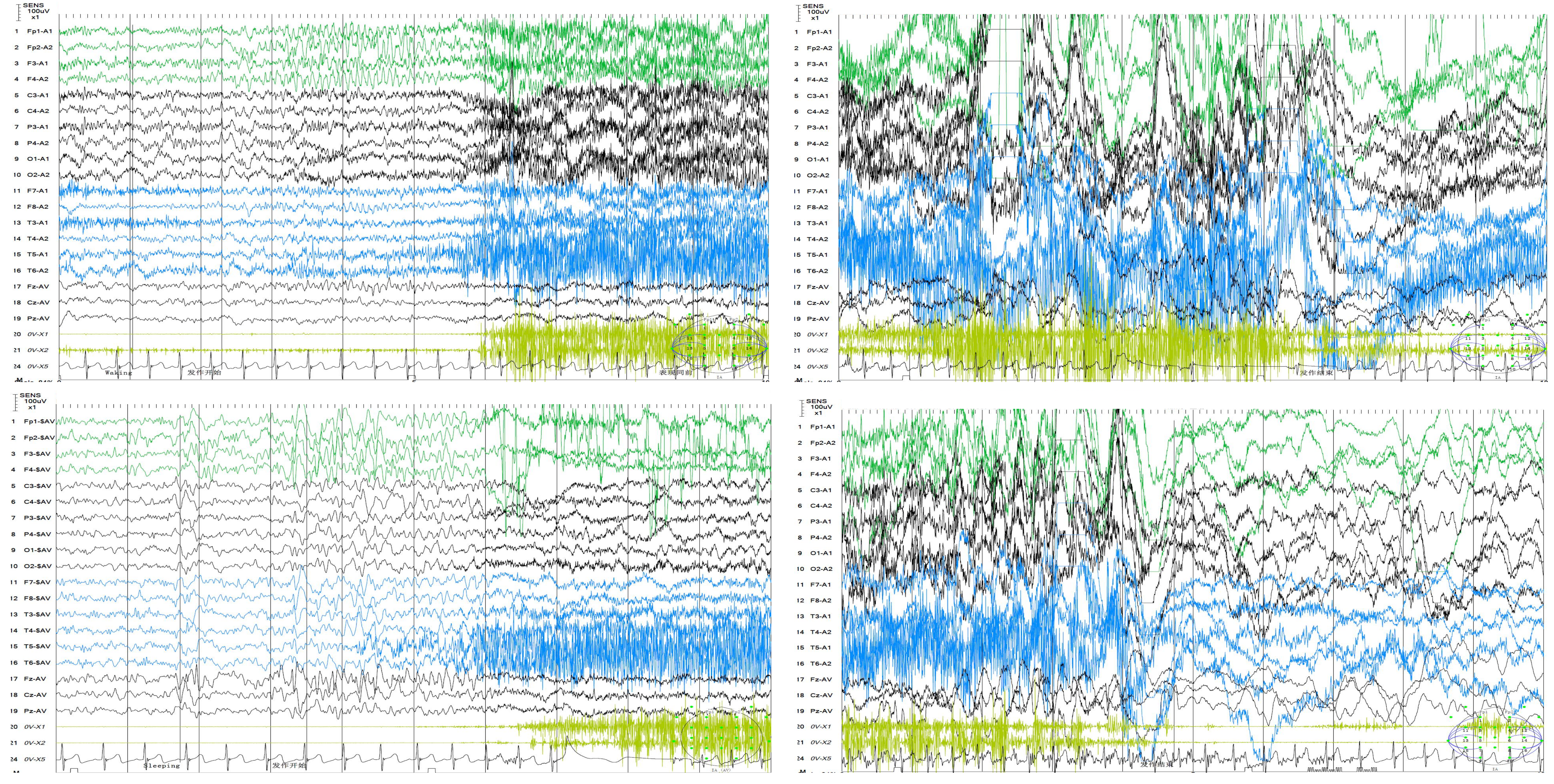


Supplementary figure 2: Preoperative ictal VEEG of case 1, showing epileptiform discharge in the right frontal area that rapidly spread to the bilateral frontal area. Extensive slow waves continued to discharge after the seizure.

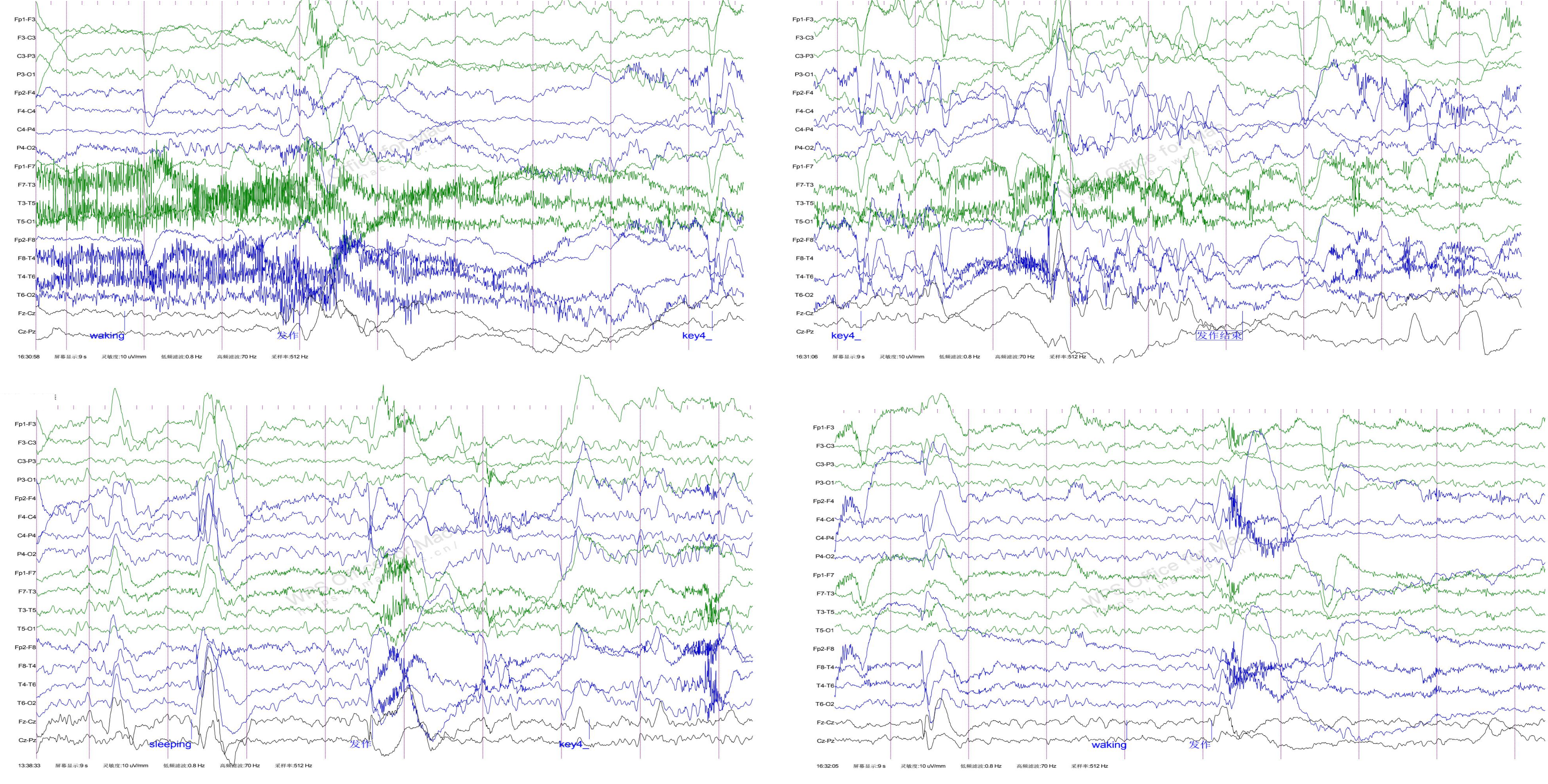


Supplementary figure 3: Preoperative VEEG of case 2. The ictal VEEG shows multiple spick (slow) waves in the right frontal, frontal, and anterior temporal regions.

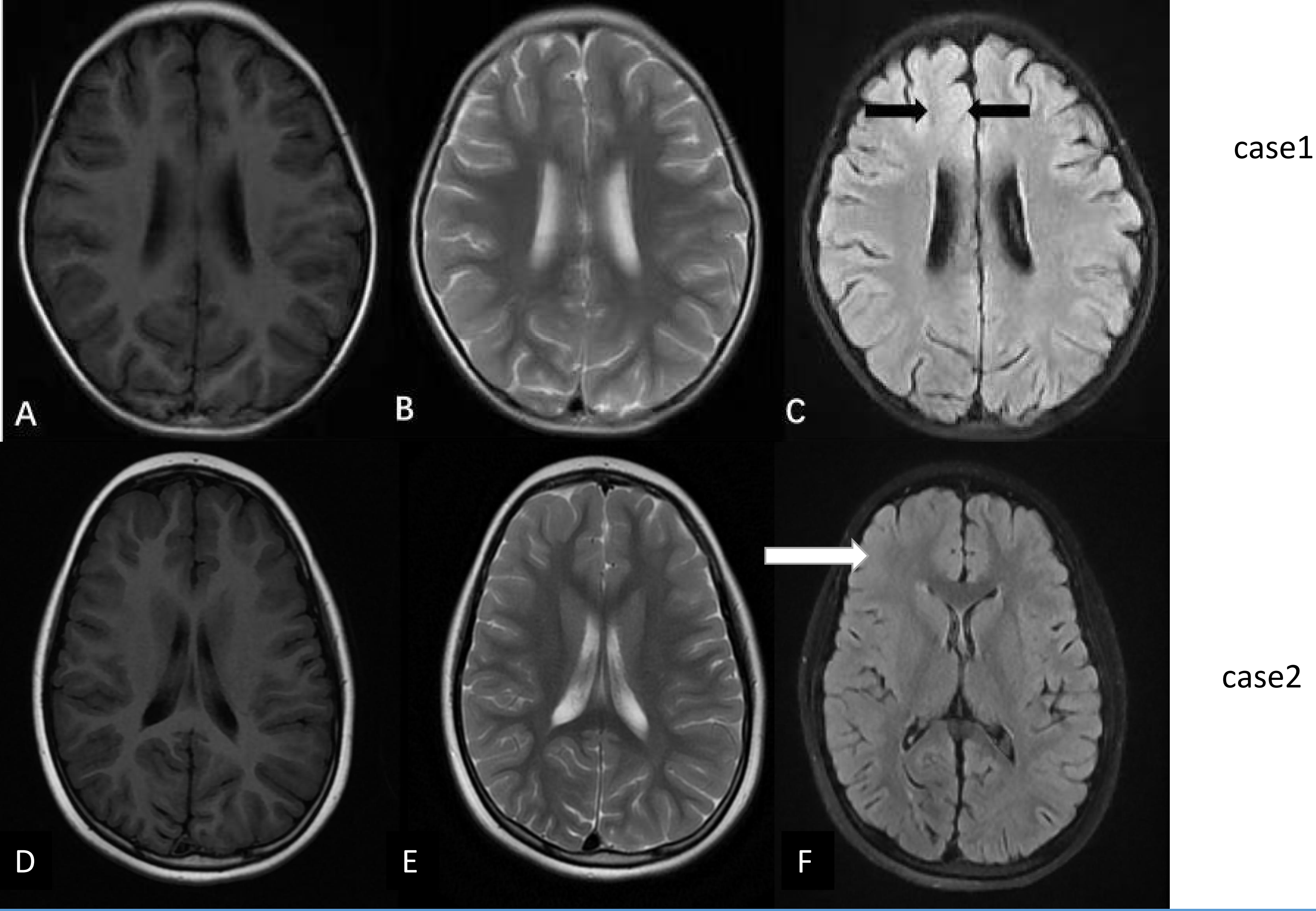


Supplementary figure 4: Preoperative head MRI of case 1: T1WI (A) and T2WI (B) sequences showed suspicious signal abnormalities, with a subtly increased FLAIR (C) signal in the right frontal lobe (black arrows). Case 2: T1WI (D), T2WI (E), and FLAIR (F) sequences showed suspicious signal abnormalities and the possibility of dysplasia of the local right frontal cortex.

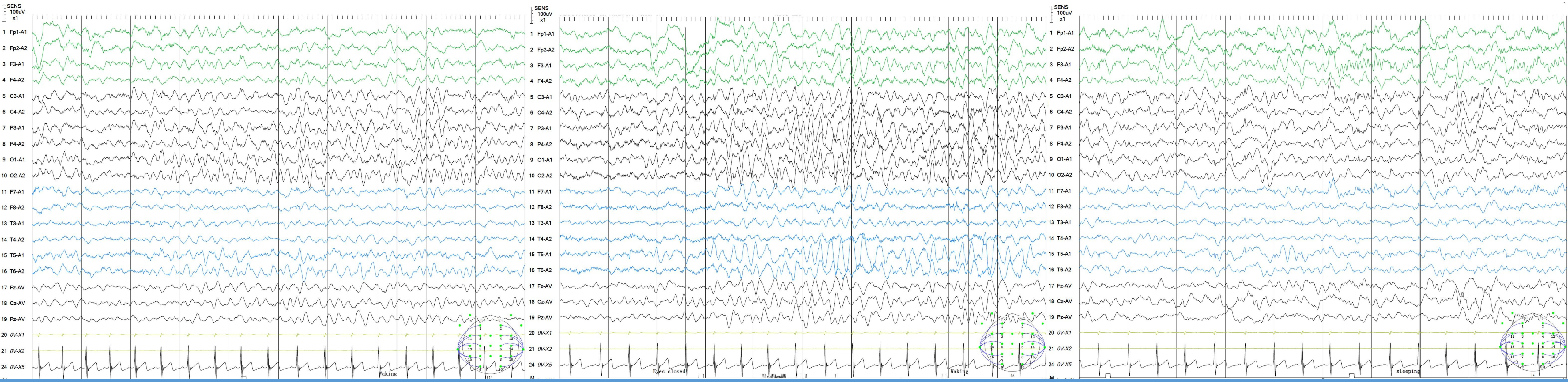


Supplementary figure 5: The postoperative VEEG of case1, displaying slow waves in the left frontal and anterior temporal regions.

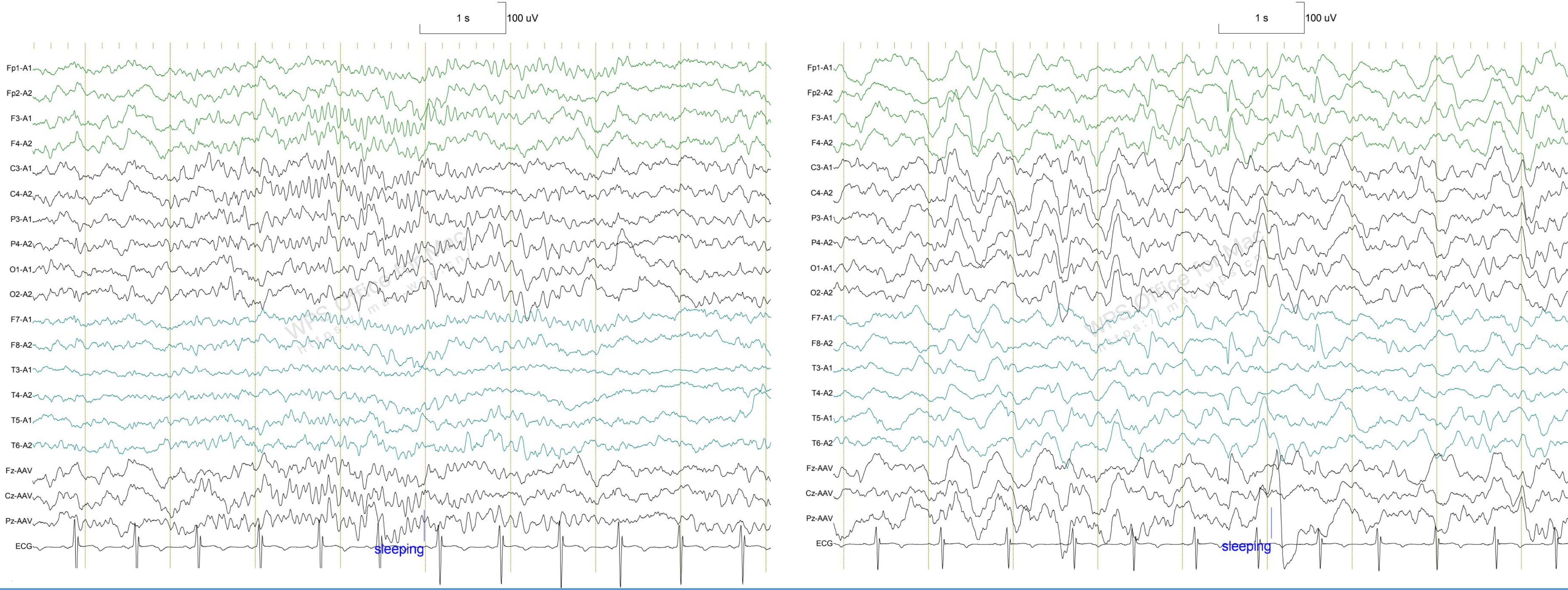


Supplementary figure 6: The postoperative VEEG of case2 showed occasional sharp waves in the forehead and frontal regions.
